# Cardiorespiratory coupling tightens with workload during graded exercise: A pilot study with adolescent athletes

**DOI:** 10.14814/phy2.70884

**Published:** 2026-04-26

**Authors:** Lizeth Avila‐Gutierrez, Eric Alonso Abarca‐Castro, Ashuin Kammar‐García, Otniel Portillo‐Rodríguez, José Javier Reyes‐Lagos

**Affiliations:** ^1^ Department of Technological Management (Biomedical Engineering) National Institute of Geriatrics (INGER) Mexico City Mexico; ^2^ School of Engineering Autonomous University of the State of Mexico (UAEMéx) Toluca State of Mexico Mexico; ^3^ Department of Biomedical Systems, School of Engineering National Autonomous University of Mexico (UNAM) Mexico City Mexico; ^4^ Division of Biological and Health Sciences Metropolitan Autonomous University, Lerma Campus (UAM‐L) Lerma State of Mexico Mexico; ^5^ Research Directorate National Institute of Geriatrics (INGER) Mexico City Mexico; ^6^ School of Medicine Autonomous University of the State of Mexico (UAEMéx) Toluca State of Mexico Mexico; ^7^ Bioelectronics Section, Electrical Engineering Department Center for Research and Advanced Studies Mexico City Mexico

**Keywords:** cardiorespiratory coupling, incremental exercise test, joint symbolic dynamics, oxygen uptake (VO_2_), ultra‐short‐term HRV

## Abstract

Cardiorespiratory coupling (CRC) reflects coordination between heart and lung function, but how it changes with increasing intensity during graded exercise remains unclear. We investigated CRC as real‐time covariation between cardiac timing and breath‐by‐breath pulmonary oxygen uptake (VO_2_) to test whether coupling strengthens with workload in adolescent athletes. We conducted an observational, within‐subject analysis of the ACTES cycling dataset. Eighteen adolescents cycled at 50, 110, and 140 W. Beat‐to‐beat RR intervals and pulmonary VO_2_ time series were examined in ultra‐short 60 s segments. Fluctuations were summarized using SDRR/RMSSD and SDVO_2_/RMSSDVO_2_. CRC was measured using joint symbolic dynamics (JSD; Shannon entropy, SE, and Miller–Madow–corrected entropy, CSE) and the highest normalized cross‐correlation (X‐Corr). Mean RR decreased and mean pulmonary VO_2_ increased with workload (both *p* < 0.0001). SDRR and RMSSD were lower at 110 and 140 W versus 50 W; SDVO_2_ declined from 50 to 110/140 W, whereas RMSSDVO_2_ was unchanged. X‐Corr increased from 50 to 110/140 W (*p* ≤ 0.0014). JSD indices decreased as workload increased (SE: global *p* = 0.139; CSE: global *p* = 0.029), suggesting tighter CRC. CRC becomes more pronounced with increased workload, aligning with reduced heart rate variability and reflecting vagal withdrawal and reflex responses that improve heart–lung integration in adolescent athletes.

## 1 INTRODUCTION

The incremental exercise test to exhaustion (GXT) is a reliable tool for examining how the body adapts to training, especially in athletes with robust cardiorespiratory fitness (Jurasz et al., 2022). During GXT, variables such as pulmonary oxygen uptake (VO_2_), blood lactate, ventilatory threshold, heart rate, carbon dioxide production, and respiratory exchange ratio are commonly assessed to characterize functional capacity and exercise performance (Bentley et al., 2007; de Sousa et al., 2021).

Physiological responses during GXT emerge from the dynamic interaction between the cardiovascular and respiratory systems as metabolic demand increases (Michael et al., 2017). Within this context, cardiorespiratory coupling (CRC) refers to the bidirectional coordination between the cardiac and respiratory systems (Cairo et al., 2025). In sports settings, CRC appears stronger in athletes than in sedentary individuals (de Abreu et al., 2023) and is shaped by both mechanical and neural mechanisms (Verhoeff & Mitchell, 2017). Specifically, CRC arises from interactions linking cardiac and respiratory activity, including respiratory modulation of vagal output and intrathoracic pressure changes that affect stroke volume and baroreflex engagement (Dick et al., 2014; Grossman & Taylor, 2007).

Pulmonary VO_2_ during exercise is a common indicator of cardiorespiratory fitness, representing the combined ability of the cardiovascular system and peripheral tissues to deliver and use oxygen throughout the body (Holmgren, 1967; Korivi et al., 2025; Myers et al., 2015). As workload increases, the cardiovascular system adjusts cardiac output and peripheral perfusion to meet the increased oxygen demand (Mezzani, 2017). Endurance training can differentially modulate these responses and influence cardiac autonomic regulation, as reflected in heart rate variability (HRV) metrics (Michael et al., 2017). Pulmonary VO_2_ measured at the mouth reflects integrated cardiorespiratory and ventilatory dynamics, motivating the examination of the relationship between cardiac timing and pulmonary gas‐exchange dynamics during exercise (Poole & Jones, 2017; Rossiter & Poole, 2023).

In this study, CRC is defined as the interaction between breath‐by‐breath pulmonary VO_2_ and cardiac timing, measured by RR‐interval fluctuations during graded exercise testing (de Abreu et al., 2023). This relationship can be analyzed using both linear and nonlinear methods, such as cross‐correlation and joint symbolic dynamics (Baumert et al., 2015; de Abreu et al., 2023; Schulz et al., 2013).

GXT protocols allow simultaneous analysis of heart rate, pulmonary VO_2_, carbon dioxide production (VCO_2_), and ventilatory equivalents (Hebisz et al., 2021; Morales‐Alamo et al., 2015). Pulmonary VO_2_max reflects the maximal integrative capacity of the lungs and cardiovascular system to support whole‐body oxygen transport and utilization during exercise (Poole et al., 2008) and is linked to cardiovascular disease risk and exercise prescription (Wang et al., 2021).

Traditional approaches, most notably HRV spectral analysis, are widely used to estimate autonomic modulation (Dong, 2016). However, their relationship with exercise capacity is inconsistent: multiple studies report no clear association between HRV indexes and pulmonary VO_2_ or pulmonary VO_2_peak (Hedelin et al., 2001; Vesterinen et al., 2015, 2016). This inconsistency supports the use of multivariate, nonlinear signal‐processing methods that can capture interactions across physiological systems. Particularly, Joint Symbolic Dynamics (JSD) may offer more profound insight into CRC (Kabir et al., 2013; Reulecke et al., 2018). Prior work has examined synchronization between cardiac and respiratory rhythms in athletes (de Abreu et al., 2022, 2023) and linked it to exercise capacity, suggesting more efficient pulmonary gas exchange and improved ventilation–perfusion matching. Such findings can inform exercise prescription (de Abreu, Cairo, et al., 2024) and support noninvasive diagnostic applications (Porta‐García et al., 2023). Recent studies also report reduced cardiorespiratory coordination during maximal incremental tests (Garcia‐Retortillo et al., 2017). More broadly, exercise profoundly reshapes human physiological regulation at multiple levels (Koay et al., 2021).

This exploratory study defines CRC as the moment‐to‐moment coupling between the RR time‐fluctuation series and the pulmonary VO_2_ series, specifically examining their temporal variability during GXT in adolescent athletes. CRC was primarily quantified using nonlinear JSD, from which Shannon entropy (SE) and corrected Shannon entropy (CSE) were derived as the primary outcome measures describing CRC dynamics. Additionally, maximum cross‐correlation was computed as a complementary linear approach to evaluate lag‐dependent relationships and provide a reference framework for interpreting the nonlinear coupling patterns. We hypothesize that CRC strengthens with increasing workload during graded exercise in adolescent athletes.

## 2 MATERIALS AND METHODS

### 2.1 Dataset description

This study is a secondary, observational, within‐subject analysis of a prospectively collected, open‐access physiological dataset. We used the “Cardiorespiratory measurement from graded cycloergometer exercise testing (GXT)” database collected in 2017–2018 at the ACTES laboratory (Université des Antilles) (Chabert et al., 2022; Mongin et al., 2021). The sample comprises 18 adolescent athletes (10 males, 8 females; 15.2 (2.0) years) from the French West Indies practicing fencing (*n* = 10), kayaking (*n* = 6), and triathlon (*n* = 2). Performance descriptors provided in the dataset include mechanical power at ventilatory thresholds (P_VT1 and P_VT2), which serve as objective indicators of aerobic performance capacity during graded exercise testing. Basic anthropometric characteristics of the participants are summarized in Table 1.

**TABLE 1:** Anthropometric and performance characteristics of the participants (*N* = 18).

Variable	Mean (SD)
Age (years)	15.2 (1.9)
Body mass (kg)	64.7 (15.5)
Height (cm)	173.3 (10.4)
BMI (kg·m^−2^)	21.2 (3.0)
P_VT1 (W)	113.0 (46.9)
P_VT2 (W)	187.3 (63.3)

*Note*: Sport distribution: fencing (*n* = 10), kayaking (*n* = 6), and triathlon (*n* = 2).

All participants completed sports‐medicine screening and a medical questionnaire and provided written informed consent with guardian authorization; the procedures were approved by the relevant ethics committees and complied with the Declaration of Helsinki. The ACTES dataset is publicly available on PhysioNet (version 1.0.0) at https://physionet.org/content/actes‐cycloergometer‐exercise/1.0.0/; no additional ethical approval was required for this secondary analysis of fully de‐identified data.

### 2.2 Experimental protocol and data acquisition (source dataset)

This work is a complementary analysis of a publicly available dataset; thus, the experimental procedures summarized below were performed in the original data‐collection study and are included here only as a brief statement of what was measured and how the signals were acquired (Chabert et al., 2022). In brief, the source study acquired beat‐to‐beat RR intervals from a 12‐lead ECG (1 ms resolution) and breath‐by‐breath pulmonary VO_2_ during a graded exercise test with stepwise workload increments to volitional exhaustion. Ventilatory thresholds (VT1 and VT2) were determined in the original ACTES dataset using the Wasserman method based on respiratory gas‐exchange variables. Briefly, VT1 was identified when the ventilatory equivalent for oxygen (VE/VO_2_) increased without a concomitant rise in VE/VCO_2_, whereas VT2 corresponded to the point where both ventilatory equivalents increased simultaneously, indicating respiratory compensation (Chabert et al., 2022). The distributed files include subject‐info.csv (demographics/performance descriptors) and test_measure.csv with beat‐indexed time, RR, VO_2_, and power; complete variable definitions and acquisition details are provided in the PhysioNet record (Chabert et al., 2022).

### 2.3 Preprocessing of RR and pulmonary VO_2_ time series

Raw RR interval series (ms) and breath‐by‐breath pulmonary VO_2_ series (L·min^−1^) were first segmented by workload. Because this was an exploratory study, we restricted the analysis to three quasi‐steady‐state workloads (50, 110, and 140 W) and to ultra‐short 60 s excerpts per workload to characterize ultra‐short‐term dynamics. For the 50 W stage, we used a fixed window from 0 to 60 s relative to the onset of that stage; for 110 and 140 W, we used the first 60 s from each stage onset, corresponding to the duration of each workload step in the GXT protocol. If a stage contained multiple contiguous blocks for a participant, each block contributed one 60 s excerpt. Segments with fewer than five data points in either signal were excluded.

RR preprocessing targeted spuriously detected intervals and ectopic beats. We used an adaptive beat‐to‐beat filter that (i) flags outliers based on local RR variability envelopes and (ii) replaces flagged beats via short‐gap interpolation to preserve timing structure, as previously described (Wessel et al., 2000). This approach mitigates artifact‐driven inflation of variability metrics while retaining physiological fluctuations.

VO_2_ time series were intentionally kept minimally preprocessed to preserve low‐frequency and direct‐current (DC; i.e., non‐oscillatory baseline) components that are physiologically meaningful during constant‐load cycling. Apart from native device quality control and the removal of missing values, no detrending was applied, such that both slow deterministic trends and breath‐by‐breath variability were retained. All computations were performed on paired, time‐aligned samples as acquired, without resampling.

### 2.4 Ultra‐short‐term RR and VO_2_ fluctuations assessment

For each participant and workload, paired RR and pulmonary VO_2_ time series were analyzed in ultra‐short 60 s segments (50, 110, and 140 W). This window length is common in exploratory/field settings and is considered acceptable for time‐domain HRV metrics (Esco & Flatt, 2014). Accordingly, we focused on standard deviation (SD) and short‐lag variability (RMSSD) for both RR and pulmonary VO_2_.

Let $x\left(t\right)$ denote a uniformly sampled series within a 60 s segment (filtered RR or pulmonary VO_2_), with N samples $\left\{x_{1},\ldots ,x_{N}\right\}$ and mean $\bar{x}=\frac{1}{N}\sum_{i=1}^{N}x_{i}$.

RRmean (mean RR): central tendency of the filtered RR series.

VO_2_mean (mean pulmonary VO_2_): central tendency of the breath‐by‐breath VO_2_ series.

Standard deviation (SD) is defined as follows:

(1) $$\mathrm{SD}\left(x\right)=\sqrt{\frac{1}{N-1}\sum_{i=1}^{N}{\left(x_{i}-\bar{x}\right)}^{2}}$$

SDRR (SD of the RR fluctuation series): overall beat‐to‐beat dispersion, integrating both slower and faster RR oscillations (i.e., total variability within the 60 s window).

SDVO_2_ (SD of the pulmonary VO_2_ fluctuation series): analogously, the total breath‐by‐breath pulmonary VO_2_ variability within the same window.

Root mean square of successive differences (RMSSD):

(2) $$\text{RMSSD}\left(x\right)=\sqrt{\frac{1}{N-1}\sum_{i=1}^{N-1}{\left(x_{i+1}-x_{i}\right)}^{2}}$$

RMSSD (Root Mean Square of Successive Differences): short‐latency beat‐to‐beat variability of RR intervals predominantly reflecting vagal modulation.

RMSSDVO_2_ (Root Mean Square of Successive Differences of Pulmonary VO_2_): Pulmonary VO_2_ analogue emphasizing successive, rapid breath‐by‐breath changes during constant‐load exercise; a pragmatic descriptor of short‐term pulmonary gas‐exchange variability rather than direct intracellular metabolic fluctuation.

### 2.5 Joint symbolic dynamics (JSD)

The nonlinear approach was used to characterize the CRC or coordinated behavior between the RR and pulmonary VO_2_ time series $\left\{x_{1,n}\right\}$ and $\left\{x_{2,n}\right\}$ of length N. The bivariate sample vector $x_{n}={\left[x_{1,n},x_{2,n}\right]}^{⊤}$ was converted into a bivariate symbol vector $s_{n}={\left[s_{1,n},s_{2,n}\right]}^{⊤}$ using Joint Symbolic Dynamics (JSD) with a binary alphabet $A=\left\{0,1\right\}$. Symbolization was based on sequential differences: increases were coded as “1” and non‐increases (decrease or tie) as “0”:

(3) $$s_{i,n}=\left\{\begin{array}{c} 1,\;\left(\;x_{i,n+1-}x_{i,n}\right)>0\\ 0,\;\left(\;x_{i,n+1-}x_{i,n}\right)\leq 0 \end{array}\right.\;i=1,2$$

Short words were then formed with a sliding window of length m=2 (step = 1 sample), yielding the univariate word set $\left\{00,01,10,11\right\}$. Pairing the simultaneous words from RR and pulmonary VO_2_ produced a 4 × 4 normalized joint distribution matrix P, whose $\left(r,c\right)$ entry contains the relative frequency of observing RR‐word $r\in \left\{00,01,10,11\right\}$ together with pulmonary VO_2_‐word $c\in \left\{00,01,10,11\right\}$, within the 60 s segment (rows: RR; columns: pulmonary VO_2_). The normalization satisfies $\sum_{r,c}P\left[r,c\right]=1$. This representation preserves robust, short‐term ordinal features while reducing sensitivity to amplitude scaling.

From the joint distribution matrix P we derived the following global index:

Shannon Entropy (SE) of the joint word distribution (bits):

(4) $$\mathrm{SE}=-\sum_{r=1}^{4}\sum_{c=1}^{4}P\left[r,c\right]\;\log_{2}P\left[r,c\right]$$

As a global index, SE is inversely related to CRC: when the coupling weakens, the joint word‐distribution matrix becomes more dispersed, and SE correspondingly rises (Reulecke et al., 2018).

### 2.6 Bias‐corrected Shannon entropy (CSE)

Because empirical entropy is downward‐biased for finite samples, we additionally report a bias‐corrected Shannon Entropy (CSE) to improve the estimation of CRC derived from JSD using the Miller–Madow term for a K‐cell distribution (K=16 here) in base‐2 units (Miller, 1955):

(5) $$\mathrm{CSE}=\mathrm{SE}+\frac{K-1}{2\;N_{\mathrm{eff}}\;\ln\;2}$$

where $N_{\mathrm{eff}}$ is the number of paired word observations (i.e., the total count used to build P).

### 2.7 Cross‐correlation (X‐Corr)

As a complementary linear index of CRC, we computed the energy‐normalized cross‐correlation between the filtered RR series $x\left[n\right]$ and the raw pulmonary VO_2_ series $y\left[n\right]$ within each 60 s segment and workload, using the full lag range and no demeaning (MATLAB xcorr(…, “coeff”)). From the resulting sequence, we extracted the maximum absolute coefficient $\rho_{\max}\in \left[0,1\right]$ as a scale‐free summary of synchronous co‐fluctuation (Shumway & Stoffer, 2017).

Single definition used:

(6) $$\rho_{\mathrm{xy}}\left(\tau \right)=\frac{\sum_{n}x\left[n\right]\;y\left[n+\tau \right]}{\sqrt{\sum_{n}x{\left[n\right]}^{2}}\;\sqrt{\sum_{n}y{\left[n\right]}^{2}}},\;\rho_{\max}=\max\;|\rho_{\mathrm{xy}}\left(\tau \right)|$$

### 2.8 Statistics

Normality was evaluated for each variable using the Kolmogorov–Smirnov (KS) test. To explore workload‐dependent differences, we used a one‐way repeated‐measures ANOVA (RM‐ANOVA); when a significant main effect was detected, we applied Tukey's post hoc test. When model assumptions were not satisfied, the nonparametric Friedman test was used instead, with Dunn's correction for pairwise multiple comparisons. In addition to *p* values, effect sizes were calculated to quantify the magnitude of workload‐dependent differences. For RM‐ANOVA, partial eta squared ($\eta_{p}^{2}$) was reported, whereas Kendall's W was used as an effect‐size estimate for Friedman tests. A two‐tailed *p* < 0.05 was considered statistically significant. All analyses were performed in GraphPad Prism 10.0.0 (GraphPad Software, La Jolla, CA, USA). Global workload effects and corresponding effect sizes are summarized in Table S1.

## 3 RESULTS

### 3.1 Ultra‐short‐term RR and pulmonary VO_2_ fluctuations

**FIGURE 1:**
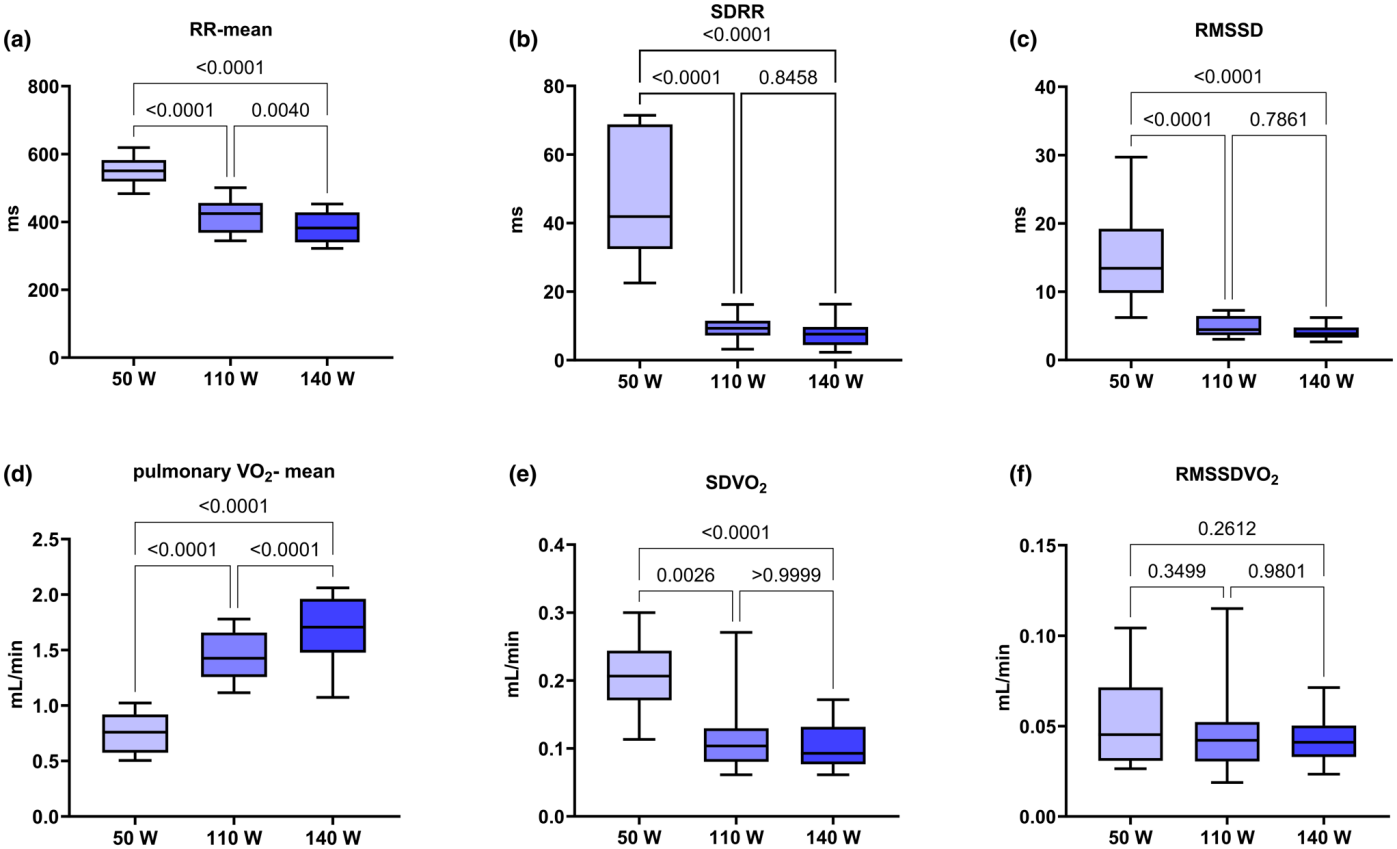
Ultra‐short‐term cardiac and pulmonary gas‐exchange metrics across quasi‐steady‐state workloads. (a–c) RR‐based indices (RR‐mean, SDRR, RMSSD). (d–f) Breath‐by‐breath pulmonary VO_2_ indices (mean VO_2_, SDVO_2_, RMSSDVO_2_). Box‐and‐whisker plots: Boxes represent the interquartile range (25th–75th percentile) with the median shown as a horizontal line; whiskers extend to the minimum and maximum observed values. Exact two‐sided pairwise *p* values are annotated above brackets in each panel. *N* = 18 per workload. Units: ms (a–c) and L·min^−1^ (d–f).

Across the three quasi‐steady‐state workloads (*n* = 18 each), cardiac timing shortened and exercise demand increased as expected (Figure 1). Data are reported as mean ± standard deviation (SD). RR‐mean decreased from 50 W (550.5 ± 38.3 ms) to 110 W (418.5 ± 48.8 ms; *p* < 0.0001) and further to 140 W (384.1 ± 46.6 ms; *p* = 0.0040) (Figure 1a). SDRR was markedly higher at 50 W (47.08 ± 17.49 ms) than at 110 W (9.47 ± 3.27 ms; *p* < 0.0001) or 140 W (7.48 ± 3.36 ms; *p* < 0.0001), with no difference between 110 and 140 W (*p* = 0.8458) (Figure 1b). RMSSD showed the same pattern: 50 W (14.91 ± 6.73 ms) exceeded both 110 W (4.93 ± 1.47 ms; *p* < 0.0001) and 140 W (4.04 ± 0.97 ms; *p* < 0.0001), while 110 versus 140 W did not differ (*p* = 0.7861) (Figure 1c).

Pulmonary gas‐exchange measures paralleled these changes. Mean pulmonary VO_2_ increased stepwise from 50 W (0.7568 ± 0.1675 L·min^−1^) to 110 W (1.451 ± 0.206 L·min^−1^; *p* < 0.0001) and from 110 to 140 W (1.683 ± 0.268 L·min^−1^; *p* < 0.0001) (Figure 1d). SDVO_2_ was greater at 50 W (0.2056 ± 0.0551 L·min^−1^) than at 110 W (0.1155 ± 0.0494 L·min^−1^; *p* = 0.0026) and 140 W (0.1043 ± 0.0329 L·min^−1^; *p* < 0.0001), with 110 versus 140 W indistinguishable (*p* > 0.9999) (Figure 1e). RMSSDVO_2_ showed no significant pairwise differences (50 vs. 110 W *p* = 0.3499; 110 vs. 140 W *p* = 0.9801; 50 vs. 140 W *p* = 0.2612), although absolute values trended slightly downward across loads (0.0515 ± 0.0227, 0.0448 ± 0.0208, and 0.0438 ± 0.0134 L·min^−1^, respectively) (Figure 1f). Global workload effects and corresponding effect sizes are provided in Table S1.

### 3.2 Cardiorespiratory coupling (CRC)

**FIGURE 2:**
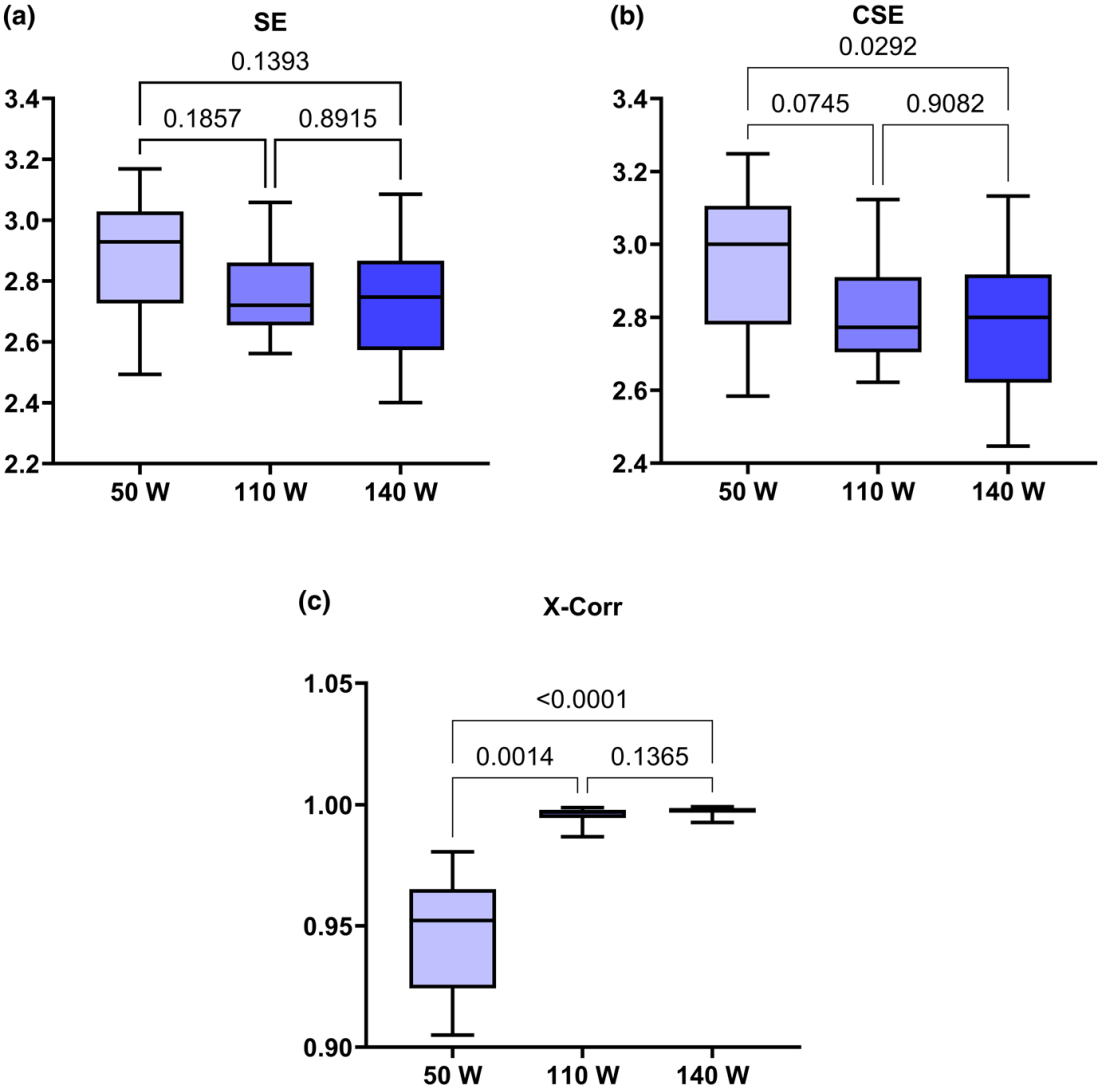
CRC metrics across workloads. (a, b) Shannon entropy (SE) and corrected Shannon entropy (CSE) derived from joint symbolic dynamics (JSD). (c) Maximum normalized cross‐correlation (X‐Corr) between RR and pulmonary VO_2_. Box‐and‐whisker plots: Boxes represent the interquartile range (25th–75th percentile) with the median shown as a horizontal line; whiskers extend to the minimum and maximum observed values. Exact two‐sided *p* values for global and pairwise tests are annotated on each panel. *N* = 18 per workload. Units are arbitrary for SE/CSE and unitless for X‐Corr.

Symbolic complexity indexes changed modestly with workload, whereas linear comovement strengthened (Figure 2). SE from joint symbolic dynamics showed an overall, nonsignificant downward trend across loads (global *p* = 0.1393), with pairwise comparisons 50 versus 110 W *p* = 0.1857 and 110 versus 140 W *p* = 0.8915 (Figure 2a); group means were 2.872 ± 0.195 at 50 W, 2.766 ± 0.148 at 110 W, and 2.748 ± 0.182 at 140 W. CSE exhibited a small overall effect (global *p* = 0.0292), driven by higher values at 50 W (2.941 ± 0.198) relative to the two higher loads (110 W: 2.817 ± 0.150; 140 W: 2.794 ± 0.185), although pairwise tests did not reach significance for 50 versus 110 W (*p* = 0.0745) or 110 versus 140 W (*p* = 0.9082) (Figure 2b). In contrast, the maximum normalized X‐Corr between RR and pulmonary VO_2_ increased robustly from 50 W (0.9467 ± 0.0242) to 110 W (0.9957 ± 0.0030; *p* = 0.0014) and from 50 W to 140 W (0.9973 ± 0.0018; *p* < 0.0001), with no difference between 110 and 140 W, *p* = 0.1365 (Figure 2c).

**FIGURE 3:**
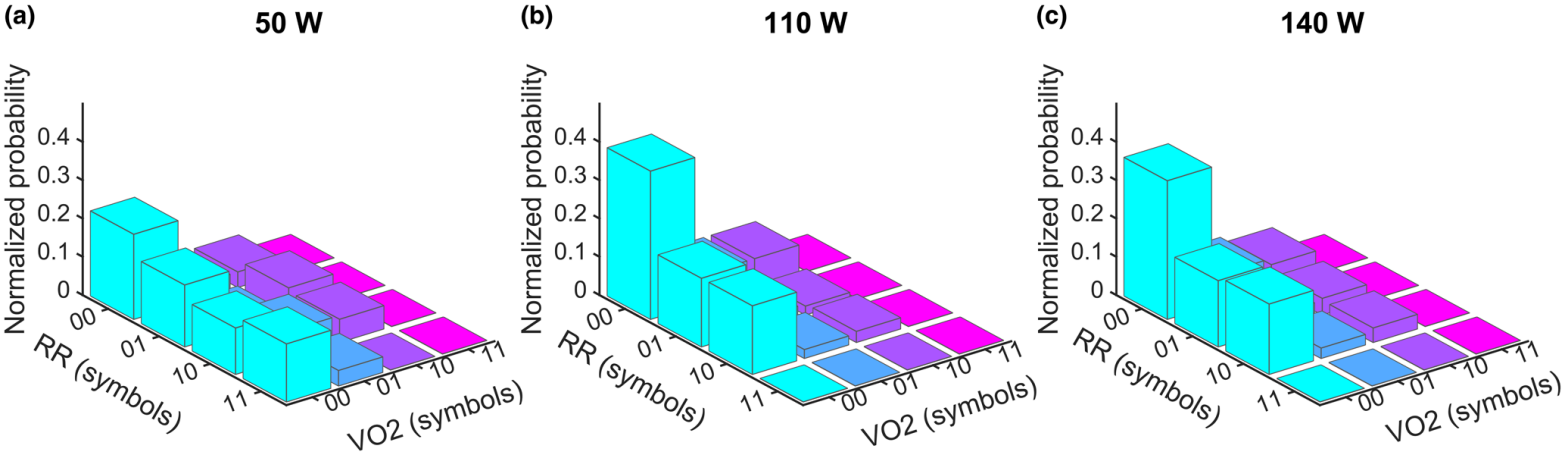
Representative joint symbolic dynamics (JSD) probability landscapes across workloads. (a)–(c) show a single participant at 50, 110, and 140 W. 3D bars depict the normalized probabilities of joint two‐symbol words for RR (front axis; codes 00–11) and pulmonary VO_2_ (right axis; codes 00–11); each tile corresponds to a specific RR–pulmonary VO_2_ word pair. Taller bars indicate more probable joint patterns. Example from one participant (ID1); group statistics are not repeated here.

To illustrate how JSD underlie the summary indices, Figure 3 shows the normalized joint word‐probability matrices for a representative participant at each workload. As load increases, probability mass concentrates into a smaller subset of RR–pulmonary VO_2_ word pairs, that is, more dominant joint patterns, visually indicating tighter co‐fluctuation. This qualitative shift is consistent with the decreases in CSE reported above (Figure 2a,b), which reflect stronger CRC as these entropy‐based indices decrease.

## 4 DISCUSSION

To our knowledge, this is among the first studies to operationalize CRC, conceived here as the moment‐to‐moment interaction between cardiac timing (RR‐interval fluctuations) and pulmonary oxygen uptake (VO_2_) during a GXT. Using ultra‐short windows, our exploratory findings suggest that CRC becomes tighter as workload increases in adolescent athletes.

As expected for incremental exercise, RR‐mean shortened and mean pulmonary VO_2_ increased with rising workloads, while short‐term and total RR variability (RMSSD, SDRR) were markedly lower at 110/140 W than at 50 W, consistent with parasympathetic (vagal) withdrawal and the characteristic reduction in HRV as exercise intensity increases from light to moderate‐high levels. Notably, no further HRV decrease was observed between 110 and 140 W, suggesting that once a moderate intensity is reached, additional increments elicit minimal HRV change, as previously reported by Michael et al. (2017), who indicated that exercise intensity is the primary factor influencing HRV, with greater intensity eliciting lower HRV up to moderate‐high levels, beyond which values tend to stabilize (Michael et al., 2017).

Breath‐by‐breath variability of pulmonary VO_2_ also declined from 50 W to 110/140 W (SDVO_2_), whereas differences between 110 and 140 W were negligible, suggesting that once cardiorespiratory and ventilatory control mechanisms are engaged, additional small load increments may alter means more than fluctuations within a 60 s window. These patterns align with the broadly linear relation between heart rate/work rate and pulmonary gas exchange over submaximal ranges (Schantz et al., 2019).

The dispersion of pulmonary VO_2_ at higher workloads should be interpreted in light of the breath‐by‐breath nature of pulmonary gas‐exchange measurements and interindividual variability in ventilatory responses. Accordingly, the coupling described here should be interpreted as a cardiorespiratory phenomenon emerging from heart–lung interaction and pulmonary gas‐exchange dynamics, rather than as a direct proxy of alveolar–capillary transfer or intracellular metabolic processes.

Two complementary CRC indices converged. X‐Corr rose from 50 W to 110/140 W, while JSD‐based entropy indices trended downward, that is, the joint word‐probability matrix became more concentrated, which in this framework denotes stronger coupling between cardiac timing and pulmonary oxygen uptake dynamics. Joint symbolic approaches are well suited to capture nonlinear coupling between physiological time series and have been recommended for the study of cardiorespiratory interactions (Baumert et al., 2015; Cairo et al., 2023). More broadly, contemporary reviews emphasize that cardiorespiratory coupling is both mechanistically and clinically meaningful in sports physiology, reflecting how neural and mechanical interactions shape performance and recovery (de Abreu, Neves, & Cairo, 2024). However, analytical approaches vary, and no single “gold standard” currently exists for quantifying these interactions (de Abreu et al., 2023). In this context, combining nonlinear symbolic metrics (e.g., SE and CSE) with a straightforward linear measure such as X‐Corr offers a complementary and robust framework for assessing CRC, integrating different aspects of the heart–lung interplay within the same physiological context. Tighter coupling may reflect greater physiological efficiency and superior aerobic performance. In this context, symbolic‐dynamics‐based analyses may provide additional insight into the regulation of heart–lung interaction during exercise.

**FIGURE 4:**
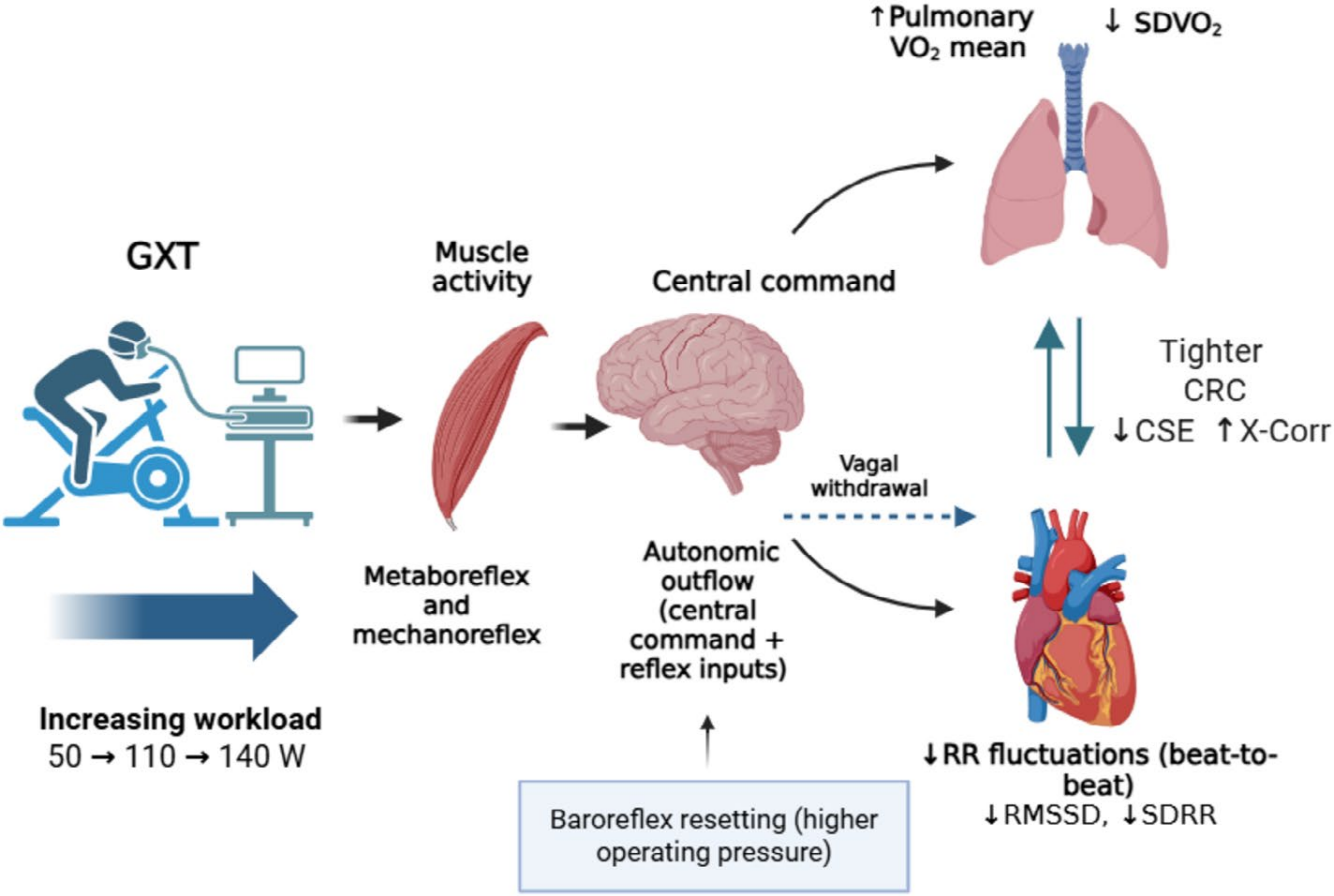
Conceptual summary of CRC during GXT. Increasing workload (50 → 110 → 140 W) engages muscle metaboreflex/mechanoreflex (group III/IV) and central command, which—together with possible baroreflex adjustment to higher operating pressures—shape autonomic outflow. Progressive vagal withdrawal (blue dashed arrow) reduces beat‐to‐beat RR fluctuations (↓RMSSD and ↓SDRR), while ventilatory and circulatory adjustments modulate pulmonary VO_2_ dynamics and reduce breath‐by‐breath variability (↓SDVO_2_). Pulmonary VO_2_ is measured at the mouth and reflects integrated cardiorespiratory and ventilatory dynamics. The resulting alignment between RR and breath‐by‐breath pulmonary VO_2_ reflects tighter CRC, summarized by lower JSD‐based entropy (↓CSE) and higher cross‐correlation (↑X‐Corr).

Physiologically, tighter CRC with increasing workload is plausible (Figure 4): (i) central command activates cardiorespiratory control centers in proportion to effort; (ii) the exercise pressor reflex (mechanoreflex/metaboreflex from group III/IV muscle afferents) reinforces sympathetic drive as exercise intensity increases; and (iii) the arterial and cardiopulmonary baroreflexes reset to operate around higher pressures, coordinating heart rate and pulmonary ventilation and gas exchange to sustain O_2_ delivery. Together, these mechanisms may produce coherent adjustments across the heart and lungs, shaping pulmonary gas‐exchange dynamics, which CRC is intended to summarize.

Importantly, our observation that RR‐based RMSSD and SDRR changes parallel CRC holds true if one considers that the initial and dominant autonomic adjustment during increasing load is vagal (parasympathetic) withdrawal, which reduces beat‐to‐beat cardiac variability while enabling tighter alignment between cardiac timing and pulmonary VO_2_ dynamics as intensity increases, a pattern also consistent with autonomic adjustments observed under psychological stress and adaptive effort regulation.

Because SE computed from finite samples is downward‐biased, we reported Miller–Madow–corrected Shannon entropy (CSE). SE showed a nonsignificant downward trend across workloads, whereas the bias‐corrected estimate (CSE) reached statistical significance. This difference likely reflects the known small‐sample bias of empirical entropy estimators, which is mitigated by the Miller–Madow correction used to compute CSE (Chen et al., 2018). In practice, X‐Corr is computationally simple and, when interpreted alongside JSD‐derived SE/CSE, may offer a robust, two‐view picture of CRC combining linear comovement and nonlinear symbolic concentration that is suitable for field and athlete‐monitoring settings (Fisher et al., 2015; Michael et al., 2017; Raven et al., 2019).

From an applied perspective, CRC metrics derived from cardiorespiratory signals may provide a compact descriptor of heart–lung integration during exercise. Such measures could potentially support athlete monitoring by indicating how efficiently cardiovascular and respiratory systems coordinate as workload increases. In addition, CRC dynamics may offer complementary information for evaluating physiological adaptation to training and could contribute to performance assessment during graded exercise testing.

At lower load (50 W), higher RMSSD/SDRR suggest stronger vagal influence and less constrained coupling. As load increases, parasympathetic withdrawal and sympathetic recruitment, driven by central command and the pressor reflex, coincide with reduced HRV and tighter CRC as the system prioritizes coordinated heart–lung adjustments to support pulmonary oxygen delivery; this coordination might be facilitated by baroreflex resetting, a mechanism previously described during exercise, although baroreflex function was not directly assessed in the present study (Fisher et al., 2015; Michael et al., 2017; Raven et al., 2019). An elevated resting CRC has been linked to higher VO_2_max during maximal exercise, indicating its potential as a marker of improved cardiopulmonary function performance (de Abreu, Neves, & Cairo, 2024).

### 4.1 Limitations and future directions

(1) Sample size was modest (*N* = 18), although the within‐subject, multi‐load design increases sensitivity to load‐dependent changes. (2) We did not stratify by sex or sport discipline; both may modulate autonomic and ventilatory responses and merit targeted study. The inclusion of athletes from different disciplines (fencing, kayaking, and triathlon) may introduce physiological heterogeneity, and discipline‐specific influences on CRC cannot be excluded. (3) We analyzed ultra‐short (60 s) windows and did not include respiratory flow or volume signals; future work should examine time‐scale dependence, test lagged JSD (to probe directionality), and integrate explicit respiratory measures, given known cardiorespiratory interactions in athletes. In the present work, CRC was assessed exclusively during steady‐state workloads. Whether similar coupling dynamics characterize transient phases, such as exercise onset, recovery, or step changes in work rate, remains an open and physiologically relevant question. Addressing these nonstationary conditions would require higher temporal resolution and dedicated analytical frameworks to capture rapid autonomic and ventilatory adjustments, which were beyond the scope of the current dataset but represent an important direction for future research. (4) Lastly, we studied trained adolescents only; therefore, no age‐matched sedentary control group was available. Consequently, it is not possible to determine whether the observed CRC tightening with increasing workload reflects athlete‐specific adaptations or a more general physiological response to graded exercise. Future studies including comparative cohorts (e.g., sedentary peers) will be necessary to address this question. Overall, our findings support CRC as a compact descriptor of heart–lung integration during incremental cycling. By combining JSD‐based entropy (SE/CSE) with X‐Corr, CRC captured a load‐dependent tightening that is mechanistically consistent with established autonomic and reflex adjustments to exercise and may hold promise for athlete monitoring and exercise prescription.

## 5 CONCLUSION

In this exploratory analysis, CRC, captured from the joint symbolic dynamics of RR fluctuations and breath‐by‐breath pulmonary VO_2_, progressively tightened with workload during graded exercise. This trend paralleled reductions in HRV indices (SDRR, RMSSD) and entropy‐based measures (SE, CSE), alongside increased linear comovement (X‐Corr), suggesting that vagal withdrawal and exercise‐related autonomic adjustments may contribute to the growing synchronization between cardiac timing and pulmonary gas‐exchange dynamics. While these findings remain preliminary, they highlight CRC as a compact, multiscale descriptor of heart–lung integration with potential relevance as a marker of physiological efficiency, adaptive stress regulation, and training adaptation.

## AUTHOR CONTRIBUTIONS

**Lizeth Avila‐Gutierrez:** Data curation; formal analysis; investigation; methodology. **Eric Alonso Abarca‐Castro:** Funding acquisition; supervision; visualization. **Ashuin Kammar‐García:** Conceptualization; formal analysis; supervision; visualization. **Otniel Portillo‐Rodríguez:** Funding acquisition; methodology; project administration; supervision. **José Javier Reyes‐Lagos:** Conceptualization; funding acquisition; investigation; supervision.

## FUNDING INFORMATION

This research received no specific grant from public, commercial, or not‐for‐profit funding agencies. Institutional support was provided by UAEMéx, INGER, UAM‐Lerma, and Cinvestav.

## CONFLICT OF INTEREST STATEMENT

The authors declare no competing interests.

## ETHICS STATEMENT

This work is a secondary analysis of an openly available, de‐identified dataset collected at the ACTES laboratory (Université des Antilles) in 2017–2018 and hosted on PhysioNet (version 1.0.0). The original data collection was approved by the relevant ethics committees, and informed consent (with guardian authorization for minors) was obtained as reported in the database documentation. The present analysis complied with the Declaration of Helsinki and applicable regulations; no new data were collected, and no re‐identification was attempted. Dataset record: https://physionet.org/content/actes‐cycloergometer‐exercise/1.0.0/.

## DECLARATION OF AI USE

AI‐assisted tools (ChatGPT) were used solely for language polishing, caption/style harmonization, and code commenting/clarification.

## Supporting information


Table S1.

Appendix S1.

## Data Availability

All raw time series used here (beat‐to‐beat RR intervals, breath‐by‐breath VO_2_, and workload annotations for 18 adolescent athletes) are publicly available on PhysioNet (see link above). The MATLAB analysis scripts used in this study—including the file‐reading utility with a user‐selectable input folder, computation of HRV metrics (meanRR, SDRR, RMSSD), VO_2_ metrics (meanVO_2_, SDVO_2_, RMSSDVO_2_), maximum normalized cross‐correlation (X‐Corr), and Joint Symbolic Dynamics entropy indices (SE and Miller–Madow‐corrected CSE)—are provided as Appendix S1.
