# Supplementary material for: Cardiorespiratory coupling tightens with workload during graded exercise: A pilot study with adolescent athletes
Source: Physiol Rep. 2026 Apr 26;14(8):e70884. doi: 10.14814/phy2.70884 (PMC13111922; doi:10.14814/phy2.70884)
Supplement: Supplementary file 1 — Table S1. [file PHY2-14-e70884-s002.docx]

**Supplementary Table S1. Global workload effects and effect sizes for RR, pulmonary VO₂, and cardiorespiratory coupling metrics**

| **Variable** | **Global test** | **Statistic** | **p value** | **Effect size** |
| --- | --- | --- | --- | --- |
| RRmean | RM-ANOVA | F(2,34)=157.0 | <0.0001 | η²p=0.902 |
| SDRR | RM-ANOVA | F(2,34)=76.48 | <0.0001 | η²p=0.818 |
| RMSSD | RM-ANOVA | F(2,34)=40.87 | <0.0001 | η²p=0.706 |
| pulmonary VO₂ mean | RM-ANOVA | F(2,34)=314.9 | <0.0001 | η²p=0.949 |
| SDVO₂ | Friedman test | χ²(2)=19.44 | <0.0001 | W=0.540 |
| RMSSDVO₂ | Friedman test | χ²(2)=3.444 | 0.1787 | W=0.096 |
| SE | RM-ANOVA | F(2,34)=3.101 | 0.0579 | η²p=0.154 |
| CSE | RM-ANOVA | F(2,34)=4.174 | 0.0239 | η²p=0.197 |
| X-Corr | Friedman test | χ²(2)=31.00 | <0.0001 | W=0.861 |

Table note: Partial eta squared (η²p) is reported for repeated-measures ANOVA, and Kendall’s W is reported for Friedman tests.
